# Synthesis of Alginate/Collagen Bioink for Bioprinting Respiratory Tissue Models

**DOI:** 10.3390/jfb15040090

**Published:** 2024-04-01

**Authors:** Amanda Zimmerling, Yan Zhou, Xiongbiao Chen

**Affiliations:** 1 Division of Biomedical Engineering, College of Engineering, University of Saskatchewan, 57 Campus Dr., Saskatoon, SK S7N 5A9, Canada; 2 Vaccine and Infectious Disease Organization (VIDO), University of Saskatchewan, Saskatoon, SK S7N 5E3, Canada; yan.zhou@usask.ca; 3 Department of Mechanical Engineering, College of Engineering, University of Saskatchewan, Saskatoon, SK S7N 5A9, Canada

**Keywords:** bioink, sodium alginate, collagen, bioprinting

## Abstract

Synthesis of bioinks for bioprinting of respiratory tissue requires considerations related to immunogenicity, mechanical properties, printability, and cellular compatibility. Biomaterials can be tailored to provide the appropriate combination of these properties through the synergy of materials with individual pros and cons. Sodium alginate, a water-soluble polymer derived from seaweed, is a cheap yet printable biomaterial with good structural properties; however, it lacks physiological relevance and cell binding sites. Collagen, a common component in the extra cellular matrix of many tissues, is expensive and lacks printability; however, it is highly biocompatible and exhibits sites for cellular binding. This paper presents our study on the synthesis of bioinks from alginate and collagen for use in bioprinting respiratory tissue models. Bioinks were synthesized from 40 mg/mL (4%) alginate and 3 mg/mL (0.3%) collagen in varying ratios (1:0, 4:1, 3:1, 2:1, and 1:1); then examined in terms of rheological properties, printability, compressive, and tensile properties and cellular compatibility. The results illustrate that the ratio of alginate to collagen has a profound impact on bioink performance and that, among the examined ratios, the 3:1 ratio is the most appropriate for use in bioprinting respiratory tissue scaffolds.

## 1. Introduction

Bioinks for bioprinting are formulations of biomaterials with bioactive components such as cells and/or growth factors which are playing an important role in the field of tissue engineering [1,2]. For this, the biomaterial must support cell attachment, proliferation, and migration and, in some cases, direct cellular differentiation, while lacking immunogenicity. From the mechanical point of view, an optimal biomaterial must also exhibit properties similar to those of the native tissue being mimicked or regenerated while also degrading at a controlled rate that matches the rate of cellular in-growth and extracellular matrix (ECM) production. Further, for use in bioprinting, the selected biomaterial must exhibit a high degree of printability which is influenced by rheological properties such as viscosity and shear-thinning behavior, as well as what crosslinking methods the material is compatible with [1,2]. Balancing all these requirements makes the development of biomaterial for bioprinting an extremely challenging process, forming one of the bottlenecks in the field of bioprinting for tissue engineering to date [3]. Combinations of hydrogel materials have been shown to exhibit a balance of these properties that allow for their use in a wide range of tissue engineering and bioprinting applications, with physiological relevance being increased with the number of different biomaterials combined; however, many biomaterials are expensive, which often becomes a limiting factor to the complexity of fabricated bioinks [4,5,6,7].

Alginate, also known as sodium alginate, or alginic acid, is a water-soluble polymer derived from seaweed [2,8]. As a natural polymer, alginate demonstrates limited immunogenicity and is biocompatible; however, it lacks cell adhesion motifs which limits its ability to be used as a biomaterial without addition of other components or other chemical modifications [9]. Due to its compatibility with ionic crosslinking, and shear-thinning behavior, alginate has been widely used in bioprinting applications, as relatively low pressures may be utilized for extrusion reducing cell death through process-induced forces, while also undergoing rapid crosslinking when printed into ionic solutions such as CaCl_2_, which allows for a high degree of printability and maintained structure fidelity over extended culture periods [10]. For example, alginate and its composites have been used in bioprinting applications spanning soft, cardiac, and bone tissues [11,12,13]. Collagens, of which there are over 20 different types, are one of the most abundant natural proteins in the body [4]. Consisting of self-aggregating polypeptide chains held together by hydrogen and covalent bonding, collagen displays a high degree of receptors for cell attachment and adhesion, and demonstrates high biocompatibility. Type 1 collagen has been used extensively in tissue engineering applications due to its physiological relevance as a structural component in tissues including respiratory, vascular, cartilage, and skin tissues, among many others [14]. Collagen is often an addition to composite bioinks as it is one of the primary ECM components in the human body (accounting for ~30% of the total body protein count). Indeed, it has been illustrated that the addition of collagen to bioinks helps support and direct natural cellular activities including cell adhesion, migration, structural stability, and tissue repair [14,15]. While collagen hydrogels can be formed though dissolution in slightly acidic solutions, and collagen can be crosslinked through control over pH and temperature, scaffolds predominantly fabricated from collagen exhibit poor printability and mechanical stability [16,17]. While collagen materials can be chemically modified to be photo-crosslinkable, it is common to combine collagen materials with other biomaterials in order to improve printability and structural fidelity [14,15,16,17,18,19].

Development of bioinks for use in respiratory tissue engineering (RTE) and bioprinting requires a bioink that mimics respiratory tissue, which is relatively soft, exhibiting stiffness values ranging from 0.44–0.75 kPA, and elastic moduli around 5–30 kPa, while also containing physiologically relevant components and exhibiting an acceptable degree of printability [20]. As type 1 collagen is the primary component of native respiratory tissue, development of collagen-based biomaterials is a promising direction of research [14]. Due to the expensive nature of collagen, as well as its lack of printability and mechanical stability, a composite hydrogel from collagen and other complementary materials should be used to synthesize the RTE bioinks. The complementary properties of alginate and collagen make them prime candidates for development of an optimized bioink for use in bioprinting respiratory tissue scaffolds [16]. Alginate and collagen have previously been combined to form inks for cartilage, cardiac, and angiogenic applications [16,21,22,23].

In this study, 4% alginate and 0.3% collagen solutions were combined in ratios including 1:0, 1:1, 2:1, 3:1, and 4:1 alginate: collagen under the hypothesis that materials with higher alginate concentrations would exhibit a greater degree of printability and mechanical strength, while materials with a greater collagen content would demonstrate greater cell viability and proliferation. Rheological studies, compression tests, tensile tests, and cell proliferation studies were all carried out to characterize the bioinks. All results were considered in terms of what would be optimal for RTE, such as the bioink exhibiting a high degree of printability, moderate mechanical properties similar to that of native lung tissue, and a high degree of cell viability.

## 2. Materials and Methods

### 2.1. Materials

Sodium alginate (medium viscosity, molecular weight~200 Kd., 180947), calcium chloride dihydrate (223506), cell proliferation reagent WST-1 (SKU5015944001), and ethylenediaminetetraacetic acid tetrasodium salt (EDTA) (E6511) were purchased from Sigma-Aldrich (St. Louis, MO, USA), while Type 1 bovine methacrylated collagen (PhotoCol Methacrylated Collagen, 9007-34-5) was purchased from Advanced Biomatrix (Carlsbad, CA, USA).

### 2.2. Bioink Synthesis

Sodium alginate (4% (*w*/*v*)) solution was prepared through dissolution of alginate powder in magnetically stirred phosphate-buffered saline (PBS) at room temperature. The solution was stirred until all powder was dissolved and a homogeneous solution was formed (~4 h). Stock collagen solution was formed through addition of 38 µL of 1.0 mol/L acetic acid solution to 33 mL distilled water. Then, 100 mg of collagen was added to the acidic solution and stirred until dissolved (~2 h). Then, the prepared 3 mg/mL collagen solution was added to the alginate solution to form the desired ratio (1:1, 2:1, 3:1, or 4:1 alginate: collagen) at room temperature. Then, 1.0 mol/L NaOH solution was added dropwise while stirring to neutralize the solution (pH = 7–7.4). Prepared solutions were refrigerated until use. All solutions were prepared twice to provide technical replicates. 

### 2.3. Rheological Properties

Rheological characterization was completed using an RVDV-X rheometer (Brookfield Engineering Labs Inc., Middleborough, MA, USA). Briefly, 2 mL of prepared uncrosslinked bioink was pipetted gently into the gap between the rheometer plate and cone, with a 0.5° geometry. Samples were set over 2 min to release any residual stress and reach the preset temperature prior to testing. Rheological properties and temperature dependence were investigated through shear stress sweep tests from 10–500 rad/s first at temperatures of 25 °C, 35 °C, and 45 °C. The Herschel–Bulkley rheological fluid-flow model was fit to each composition for analysis using MATLAB R2023A (Equation (1)).

(1) $$\tau =\tau_{0}+k{\dot{y}}^{n},$$

where τ is shear stress (Pa), τ_0_ is yield stress, k is the consistency index (Pa·s^n^), $\dot{y}$ is shear rate (s^−1^), and *n* is a dimensionless constant indicating the flow index. The determination of temperature dependence and rheological properties was later used to inform the selection of printing parameters including printing head speed, printing pressure, and temperature of the printing bed and nozzle. All studies were carried out in triplicate.

### 2.4. Printability

Initial printing parameters (printing head speed and printing pressure) were selected and modified as required to obtain a well-defined extrusion printed structure using a GeSiM mbH BioScaffolder 3.2 outfitted with a 27-gauge needle (0.21 mm inner diameter) at 25 °C. Two-layer 10 × 10 mm constructs were printed from each material composition while printing pressure and printing head speed were varied. A designed strand diameter equal to the dimension of the inner diameter of the extrusion needle used (0.21 mm) and a strand spacing of 1.0 mm were selected. Printed constructs were then imaged with strand diameter and strand spacing, among other printed dimensions being measured and recorded.

While the materials were evaluated for how closely they were able to meet the design criteria, the major goal was to select printing speeds and pressures for each material that provided the most similitude between scaffolds printed from each material. Therefore, printability was assessed in terms of selecting parameters for each material to obtain a consistent scaffold with strand diameters of 400 µm as this was a more feasible strand diameter to obtain with the alginate/collagen materials without using a smaller needle size that would cause significant cell damage during extrusion. Scaffolds were printed into a 50 mM CaCl_2_ crosslinking solution and crosslinked overnight before imaging to allow for consistent swelling effects. Compositions with acceptable printability and mechanical properties were then used for tensile testing and biocompatibility studies.

### 2.5. Compression Testing

Bulk material specimens were prepared and used for compression testing using a BOSE BioDynamic 5010 Mechanical Tester (20 N load cell capacity), Bose Corporation, Framingham, MA, USA. Briefly, each material was poured into a cylindrical mold with a diameter of 10 mm and immersed in 100 mM CaCl_2_ crosslinking solution. Molds remained immersed in the crosslinking solution until homogeneous crosslinking of the bulk constructs had been achieved (~48 h). The cylindrical bulk specimens were then removed from the mold, and sectioned into discs with a height of 5 mm for final cylindrical sample dimensions of 10 mm by 5 mm. Each specimen was measured and exact height and diameter were recorded. Specimens (n = 6) were then compressed at a rate of 0.01 mm/s to a max compression of 2 mm to ensure the safety of the load cell. The height and diameter of each specimen after compression were then measured. These static compression tests were used to determine the influence of alginate/collagen ratio on the bulk compressive modulus, and modulus of resilience.

### 2.6. Tensile Testing

Specimens were designed based on previous studies [24], for tensile testing of printed hydrogel constructs. Briefly, 3 mm tall cylindrical scaffolds with a diameter of 9 mm were printed with 1 mm strand spacing on all but the top two layers. The top two layers were printed with a strand spacing of 0.27 mm to form a solid surface. These samples were fabricated using the optimal printing parameters found in the printability study in order to print highly similar scaffolds from each material. Scaffolds were then crosslinked for 24 h in 50 mM CaCl_2_. Constructs (n = 6) underwent tensile testing with a crosshead speed of 50 µm/s to a maximum displacement of 5 mm using a previously developed tensile testing apparatus [24]. These settings were selected due to previous studies determining that they are optimal for consistency of results using this apparatus. Displacement and force measurements were collected and converted to stress–strain curves which were then used to determine Young’s modulus and ultimate tensile strength (UTS). 

### 2.7. Cellular Viability

Biocompatibility of the various inks was assessed through incorporation of human pulmonary lung fibroblasts (HPFs) (cryopreserved, C12360, LOT #446Z031), isolated from healthy human respiratory tissue, purchased from PromoCell (Heidelberg, Germany) into the bulk materials. Each material was loaded with 2 × 10^6^ primary human pulmonary fibroblasts per mL, and 100 µL of a specified material was pipetted into each well of a 96-well plate with one row (n = 12) for each material of interest. Crosslinker (50 mM CaCl_2_) was added to each well and left for 20 min before it was removed and replaced with cell culture media (Fibroblast Growth Media 2, PromoCell). The plates were cultured in an incubator (37 °C, 5% CO_2_), with a plate removed after 1, 3, 5, 7, 10, and 14 days. The cell-containing material was then dissolved with EDTA. The plates were centrifuged and the EDTA was removed and replaced with PBS. WST-1 was then added and the plates were incubated for 1 h before absorbance measurements were taken using a BioRad xMark microplate spectrophotometer (Bio-Rad, Hercules, CA, USA) at a wavelength of 440 nm.

### 2.8. Statistical Analysis

Statistical analysis was carried out using SPSS 28 statistical analysis software. Nonparametric independent samples Kruskal–Wallis tests with pairwise comparisons were utilized for the mechanical studies, with *p* < 0.05 considered significant, while general linear modeling with univariate analysis of variance (ANOVA) was used for analysis of the cellular viability and proliferation results, with *p* < 0.05 considered significant.

## 3. Results

### 3.1. Rheological Properties

**Figure 1:**
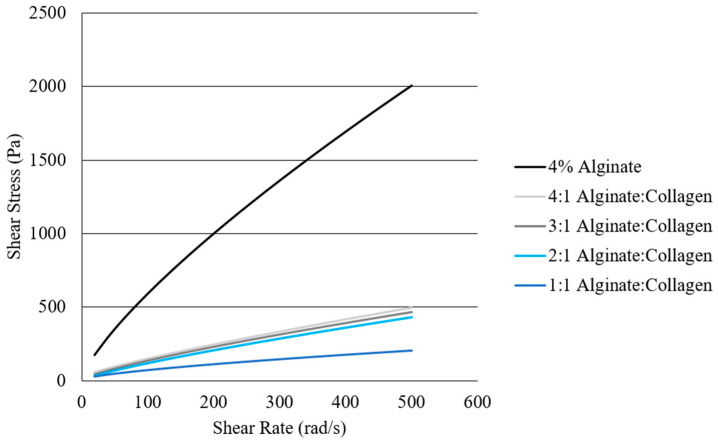
Comparison of rheological flow properties of 4% alginate, 4:1 alginate: collagen, 3:1 alginate: collagen, 2:1 alginate: collagen, and 1:1 alginate: collagen when undergoing a shear rate sweep from 10 to 500 rad/s at 25 °C.

Solutions of 1:1, 2:1, 3:1, 4:1, and 1:0 alginate: collagen underwent rheological testing to help inform the selection of printing parameters (Figure 1). 

As shown in Figure 1, viscosity decreases with an increased ratio of collagen solution, with pure 4% alginate demonstrating significantly more viscous behavior, and the 1:1 ratio demonstrating the lowest viscosity. Table 1 demonstrates the values obtained from fitting the Herschel–Bulkley fluid model to the rheological data.

In many cases, the value of τ_0_ was negligible or insignificant. All materials demonstrated shear-thinning behavior (n < 1) and were well characterized by the Herschel–Bulkley or power law models as indicated by an R^2^ of 0.99 or higher. While the consistency index (K) of all solutions containing collagen were quite similar, the 4% alginate material had a significantly higher consistency index value. 

**Figure 2:**
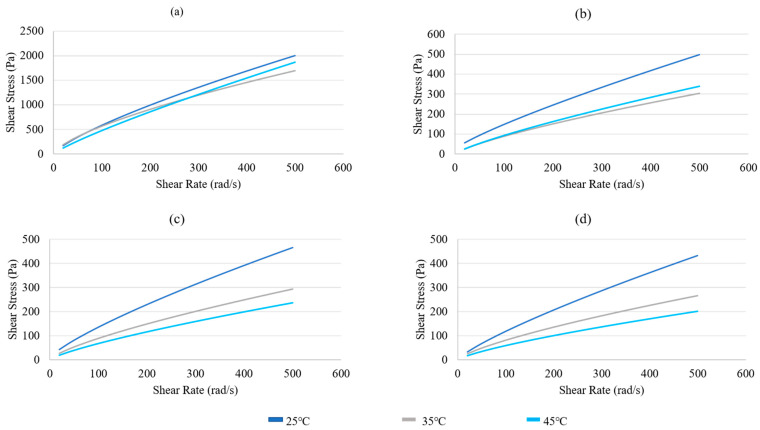
Rheological properties determined by a shear stress sweep from 10–500 rad/second at temperatures of 25, 35, and 45 °C for various materials: (**a**) 4% alginate; (**b**) 4:1 alginate: collagen; (**c**) 3:1 alginate: collagen; (**d**) 2:1 alginate: collagen.

Viscosity dependence on temperature for each material was then analyzed through shear stress sweeps at temperatures of 25, 35, and 45 °C (Figure 2). In general, viscosity decreased with increasing temperature, as expected, due to the thermal sensitivity of both alginate and collagen, with thermal crosslinking being one of the primary means of stabilizing collagen solutions [14]. The Herschel–Bulkley constant values can be found in Supplementary Table S1. The 1:1 ratio did not have a high enough viscosity to increase torque beyond 10%, which is the accuracy limit of the equipment used; therefore, further rheological data for the 1:1 alginate: collagen material are not reported here.

### 3.2. Printability

**Figure 3:**
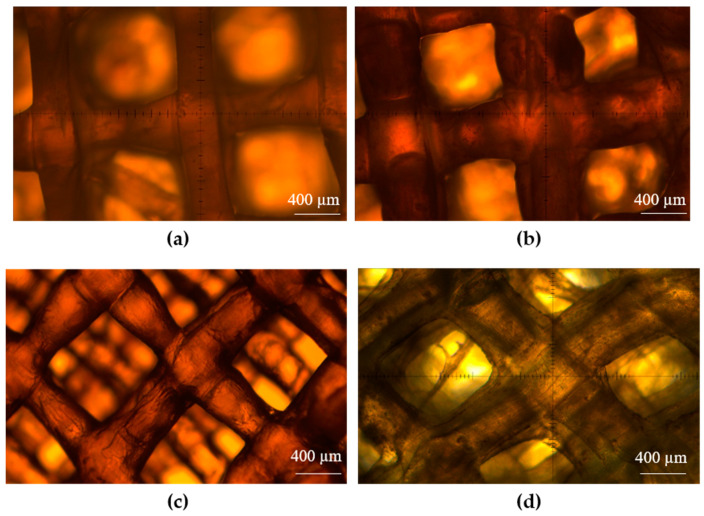
Printability assessment of various material ratios: (**a**) 4% alginate; (**b**) 4:1; (**c**) 3:1; (**d**) 2:1 alginate: collagen.

As can be seen in Figure 3, scaffolds were successfully printed from all materials with the exception of the 1:1 alginate: collagen solution. Although strands with printed diameters around 400 µm could be printed from the 1:1 material (Table 2), due to its low viscosity, the consistency of structurally stable strands was limited, leading to many gaps in the scaffold, and this limited the ability to form consistent multilayer constructs. Due to this, the 1:1 ratio was removed from consideration. In all other materials, consistent porosity and strand diameters were achieved.

Printing optimization determined that use of a 200 µm needle was suitable for 4% alginate and 4:1 alginate: collagen; however, the fast speed required for printing the 4:1 ratio material with the 200 µm needle begin to cause disruptive movement in the crosslinking media. Therefore, instead of increasing print speed further, a smaller diameter needle (150 µm) was used for all less-viscous ratio solutions. It was found that by adjusting the print speed and pressure for each material, consistent strands with diameters around 400 µm could be obtained.

### 3.3. Compression Testing

**Figure 4:**
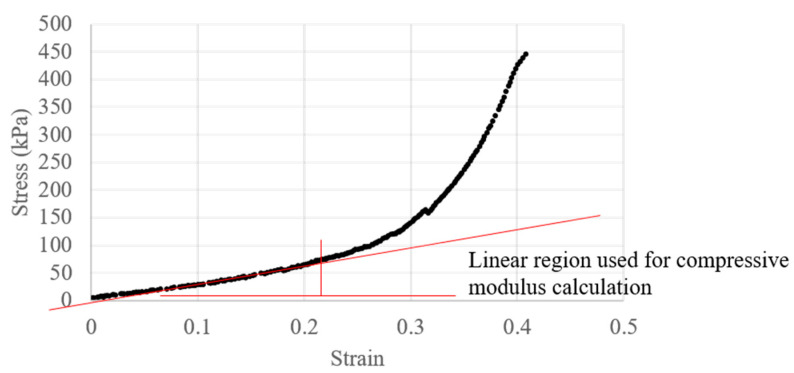
Compressive stress–strain curve of 4% alginate, used to calculate the static compressive modulus.

Figure 4 demonstrates a representative stress–strain curve obtained from the force displacement data obtained through compression testing. 

**Figure 5:**
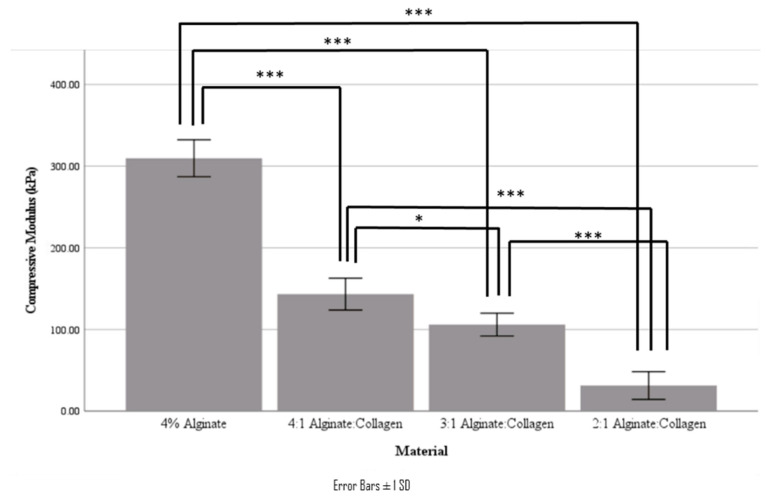
Compressive modulus of the various bulk materials (* indicates *p* < 0.05, *** indicates *p* < 0.001).

As demonstrated in Figure 5 and recorded in Table 3, as collagen concentration increases, the compressive modulus decreases, with very large decreases being seen moving from 4% alginate to 4:1 alginate: collagen, as well as between 3:1 alginate: collagen and 2:1 alginate: collagen.

### 3.4. Tensile Testing

**Figure 6:**
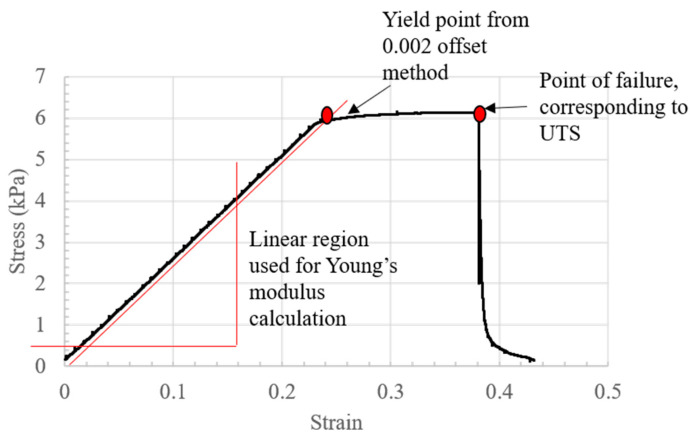
A representative stress–strain curve obtained from 3:1 alginate: collagen.

Stress–strain curves were calculated from the obtained force–displacement data. A representative stress–strain curve can be seen in Figure 6. 

**Figure 7:**
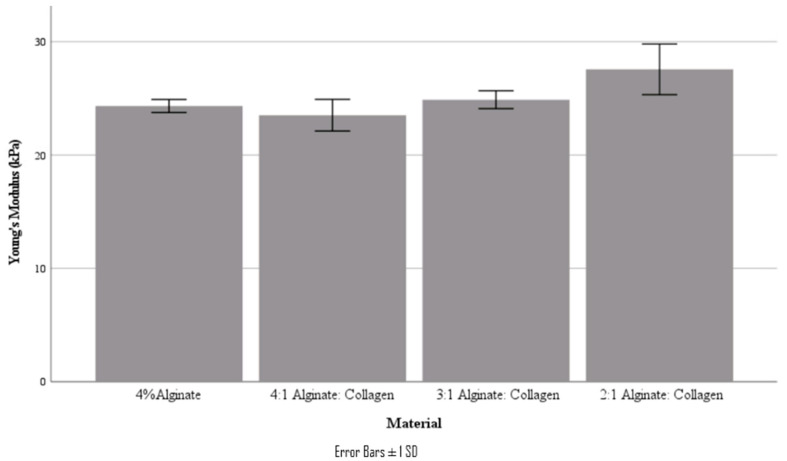
Young’s modulus of various material concentrations.

**Figure 8:**
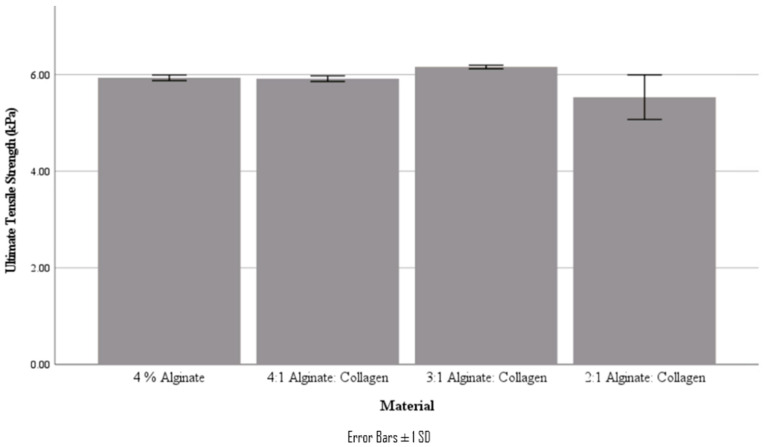
Ultimate tensile strength of various material concentrations.

From the stress–strain curves obtained, Young’s modulus and ultimate tensile strength were obtained. It should be noted that these tensile tests are characterizing the bond strength between printed layers, not the strength of a bulk material. These values are summarized in Table 4 and Figure 7 and Figure 8.

Interestingly, the tensile mechanical properties of the various ratio materials did not follow the decreasing trend that was found in the compressive studies; in fact, there was no statistically significant difference in either Young’s modulus or UTS for all materials (*p* < 0.05). It was also found that the yield point of the tested scaffolds was at a very similar stress to the UTS, as can be seen in the stress–strain curve.

### 3.5. Cellular Viability

**Figure 9:**
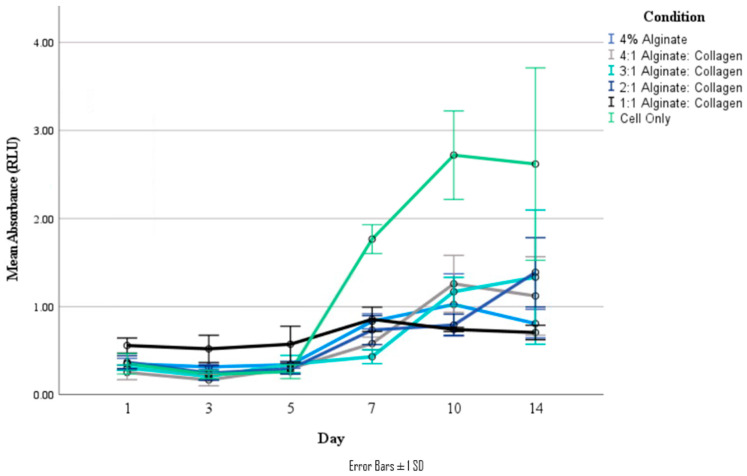
Absorbance measurements of live cell staining of various ratio materials at timepoints of 1, 3, 5, 7, 10, and 14 days.

From Figure 9, there was an initial decrease in live cell population from Day 1 to 3 before cell proliferation picked up. Both the 1:1 ratio and 4% alginate materials demonstrated a decrease in cell viability after Day 7 and Day 10, respectively, while the 3:1 and 2:1 materials demonstrated a consistent increase in cell viability after Day 5. The cell-only wells did demonstrate a significantly greater cell viability and appeared to reach confluency around Day 10, likely due to a lack of change in the culture environment, compared to encapsulating cells needing to remodel their new environment for preferable cell growth conditions [2].

## 4. Discussion

While alginate and collagen composite bioinks have been used throughout the field of tissue engineering, few studies have been reported characterizing alginate and collagen composite materials in depth, and no studies have utilized the exact same ratios of materials. Although this makes direct comparisons with the literature challenging, general trends can be seen, including good mechanical stability and a high degree of biocompatibility [25,26,27]. Furthermore, our study illustrates the promise of this composite bioink for application in RTE.

Based on the Herschel–Bulkley fluid model, it could be seen that increasing the ratio of collagen solution to alginate led to a decrease in viscosity, with a 1:1 ratio leading to a very low viscosity material. However, all material solutions demonstrated shear-thinning behavior (n < 1) which is optimal for bioprinting due to the reduced force required for extrusion. As most of the ꚍo values were minimal, the power law fluid-flow model would also be an accurate model to describe the flow behavior of alginate/collagen materials. Viscosity of each material could also be seen to generally decrease with increased temperature. The determined variation in viscosity with both collagen concentration and temperature was then used to inform the selection of starting printing parameters for each material, and a consistent printing temperature of 25 °C. In the literature, the development of an injectable alginate/collagen composite has been reported, with the results demonstrating similar trends in both alginate-concentration dependance [28] and temperature dependence [29].

Printability characterization was carried out by varying both printing pressure and speed for each material, in order to form consistent dimensions. A goal strand diameter of 400 µm was selected as it allowed the printing of multilayer (10+) scaffolds with good structural fidelity. While strands with diameters more similar to the inner diameter of the needles used (200 µm or 150 µm) could be formed by further increasing the print speed and decreasing print pressure, it was found that there were often more breaks or inconsistencies in the strands printed in these conditions. This form of strand breakage was very common in the 1:1 ratio material even at settings leading to strand diameters around 400 µm, making it unsuitable for printing larger multilayer structures which led to its removal from consideration from further experiments. Suitable settings were found for all other remaining materials and these settings were then utilized for all further printing, with printability assessments carried out at various stages through printing to ensure the maintenance of similar-diameter strands over a printing period. By maintaining consistent strand diameters between the different ratio materials, the connection area between strand layers and overall volume of material was able to be kept consistent in order to allow for direct comparison of mechanical properties of each material. While using different concentrations of their base alginate and collagen solutions, results published in the literature have also found that higher alginate concentrations tend to lead to improved printability, which is caused by the increased speed of ionic crosslinking of alginate in comparison to the crosslinking mechanisms that are compatible with collagen [21,25,30]. 

Compressive properties of each bulk material were assessed, with the compressive moduli decreasing with increasing concentration of the collagen solution. As the structural stability of the material is reliant upon the ionic crosslinking of the alginate solution, this trend is expected. However, in contrast, tensile testing of 3D printed scaffolds from each material demonstrated an increase in tensile properties moving from the 4% alginate material to the 3:1 ratio solution, while there was no significant difference in Young’s modulus. As these measured tensile properties are heavily dependent on scaffold layer bonding rather than the bulk material properties, bonding between strands with lower alginate concentrations could be greater due to a reduced rate and/or a lesser degree of ionic crosslinking leading to strands being less solidified by the time the next layer is printed on top. More studies would be required to confirm this hypothesis; however, it is also possible that collagen fibers, aligned by printing, may also provide strength when placed under tensile stress, with this ability being compromised once the main alginate structure becomes too weak to provide a stable frame. Other studies on the mechanical properties of alginate/collagen bioinks have also found increases in the tensile properties of alginate ink with the addition of collagen, as well as a similar increase in compressive properties, which might be attributed to the compact and interconnected 3D network formed through the addition of collagen [21]. 

As native lung tissue has stiffness values ranging from 0.44–0.75 kPA [20], and elastic moduli around 5–30 kPa, an optimal biomaterial would exhibit properties within these ranges, with middle range values preferred for mimicry of the native bronchioles. While the measured Young’s modulus fit into the upper range, the ultimate tensile strength was much greater than that of native lung tissue. However, as it is expected that the mechanical properties of these materials would degrade over time, alginate/collagen solutions appear to provide a good starting point, as materials with less ultimate tensile strength would be extremely difficult to handle without damaging. 

While the cell viability results show that using cells in culture media results in greater cellular proliferation over a 14-day period than cells encapsulated in the biomaterials, significant cellular proliferation can be seen in all the biomaterials after a short adaption period. This biomaterial encapsulation also likely slows cell growth and proliferation as it lessens the free movement of waste and nutrients to and from cells [2]. Over the period of 14 days, materials with higher alginate concentrations (4% alginate, and the 4:1 ratio) begin to show a decrease in cellular viability, while the 3:1 and 2:1 ratios demonstrate sustained growth. Previous studies have shown that an increase in collagen in proportion to alginate in alginate/collagen composite hydrogels tends to exhibit a greater degree of cellular viability due to the greater number of cell-binding ligands [25,26,27]. The 1:1 ratio, tested here to determine if the lesser viscosity is more favorable, also demonstrated lesser cell viability and proliferation over the 14-day time period, though this is likely due to loss of cell-containing material due to a lack of mechanical stability. The delay in increasing cell viability seen in this study appears to be similar to other studies utilizing similar materials, where significant increases are not immediately seen [26,27,29]. This is likely due to cells acclimating to their encapsulating environment, which necessitates remodeling of the environment before cell growth is promoted. 

## 5. Conclusions

Bioink synthesis is one key in the 3D printing of tissues as the biomaterial must support cell attachment and proliferation, exhibit mechanical properties similar to the targeted tissue, and have a high degree of printability in order to form consistent printed structures. Sodium alginate is a very commonly utilized biomaterial due to its cost, biocompatibility, printability, and structural fidelity, while collagen is commonly used as it is the most common natural occurring component in ECM, providing cell attachment motifs and increasing physiological relevance. In the present study, we synthesized bioink from 4% alginate and 3 mg/mL collagen in ratios of 4:1, 3:1, 2:1, and 1:1, respectively, and then characterized these bioinks in terms of rheological properties, printability, compressive and tensile properties, and cellular compatibility. Our results demonstrated that all bioinks with exception of the 1:1 ratio had acceptable printability, compressive, and tensile properties, and that the 3:1 and 2:1 ratios exhibited improved cellular proliferation. Due to the similitude in mechanical properties and biocompatibility of the bioinks with 2:1 and 3:1 ratios, the selection of the optimal material came down to cost. As collagen is a very expensive material, the bioink with a ratio of 3:1 ratio was determined to be the preferred bioink out of the tested materials for further development of 3D bioprinted lung tissue models. 

## Figures and Tables

**Table 1:** Herschel–Bulkley fluid-flow model constants and goodness of fit analysis at 25 °C (* indicates significant differences from other materials at *p* < 0.01).

Material	τ_0_	*K*	*n*	R^2^	RMSE
4% Alginate	1 × 10^−6^ ± 1 × 10^−6^	17.5 ± 7.3 *	0.8 ± 0.1	0.99	39.8
4:1 Alginate: Collagen	6 × 10^−3^ ± 3 × 10^−3^	2.7 ± 1.4	0.8 ± 0.1	1.00	2.8
3:1 Alginate: Collagen	6.7 ± 7.5	3.4 ± 1.4	0.8 ± 0.1	1.00	3.2
2:1 Alginate: Collagen	1 × 10^−2^ ± 1 × 10^−3^	2.9 ± 0.9	0.8 ± 0.1	1.00	3.5
1:1 Alginate: Collagen	6.4 ± 9.0	2.7 ± 2.0	0.7 ± 0.1	1.00	2.6

Abbreviations: τ_0_ (yield stress), *K* (consistency index), *n* (flow behavior index), R^2^ (coefficient of determination), RMSE (root-mean-square deviation).

**Table 2:** Optimal printing parameters for each material to obtain strand diameters around 400 µm.

Material	Pressure (kPa)	Speed (mm/s)	Needle Diameter (µm)	Printed Strand Diameter (µm)
4% Alginate	10	24	200	412.4 ± 17.3
4:1 Alginate: Collagen	10	47	200	406.9 ± 18.5
3:1 Alginate: Collagen	10	8	150	394.6 ± 21.5
2:1 Alginate: Collagen	9	10	150	400.3 ± 24.3
1:1 Alginate: Collagen	8	10	150	399.5 ± 27.8

**Table 3:** Compressive modulus of the various material ratios.

Material	Static Compressive Modulus (kPa)
4% Alginate	310 ± 23
4:1 Alginate: Collagen	143 ± 20
3:1 Alginate: Collagen	79 ± 20
2:1 Alginate: Collagen	31 ± 17

**Table 4:** Tensile properties of the various material ratios.

Material	Young’s Modulus (kPa)	Ultimate Tensile Strength (kPa)
4% Alginate	24.3 ± 0.6	5.93 ± 0.06
4:1 Alginate: Collagen	23.5 ± 1.4	5.92 ± 0.06
3:1 Alginate: Collagen	24.9 ± 0.6	6.16 ± 0.03
2:1 Alginate: Collagen	27.6 ± 2.2	5.53 ± 0.46

## Data Availability

All data collected and analyzed in this manuscript is available upon reasonable request from the corresponding author.

## Supplementary Materials

The following supporting information can be downloaded at: https://www.mdpi.com/article/10.3390/jfb15040090/s1, Table S1: Herschel–Bulkley Fluid-flow model fits for all rheological tests.
