# Supplementary material for: Synthesis of Alginate/Collagen Bioink for Bioprinting Respiratory Tissue Models
Source: J Funct Biomater. 2024 Apr 1;15(4):90. doi: 10.3390/jfb15040090 (PMC11050917; doi:10.3390/jfb15040090)
Supplement: Supplementary file 1 [file jfb-15-00090-s001.zip › jfb-2912717-supplementary.pdf]

Journal of
Functional Biomaterials

MDPI

Supplementary Material

Synthesis of Alginate/Collagen Bioink for Bioprinting Respiratory Tissue Models

Amanda Zimmerling [1,2,*], Yan Zhou [2] and Xiongbiao Chen [1,3,*]

[1] Division of Biomedical Engineering, College of Engineering, University of Saskatchewan, 57 Campus Dr., Saskatoon, SK S7N 5A9, Canada
[2] Vaccine and Infectious Disease Organization (VIDO), University of Saskatchewan, Saskatoon, SK S7N 5E3, Canada; yan.zhou@usask.ca
[3] Department of Mechanical Engineering, College of Engineering, University of Saskatchewan, Saskatoon, SK S7N 5A9, Canada
* Correspondence: asz694@usask.ca (A.Z.); xbc719@usask.ca (X.C.)

Table S1. Herschel-Bulkely Fluid flow model fits for all rheological tests.

Material	τ_o	*K*	*n*	R^2	*RMSE*
4% Alginate 25°C	1.3E-06	17.5	0.8	0.99	39.8
4% Alginate 35°C	2.2E-04	24.8	0.7	1.00	18.8
4% Alginate 45°C	6.8E-06	9.6	0.8	1.00	26.7
4:1 Alginate: Collagen 25°C	5.9E-03	2.7	0.8	1.00	2.8
4:1 Alginate: Collagen 35°C	2.4E-07	2.7	0.8	1.00	3.7
4:1 Alginate: Collagen 45°C	1.6E-07	2.3	0.8	1.00	3.3
3:1 Alginate: Collagen 25°C	6.7E+00	3.4	0.8	1.00	3.2
3:1 Alginate: Collagen 35°C	1.1E-08	2.9	0.7	1.00	4.2
3:1 Alginate: Collagen 45°C	7.2E-08	1.9	0.8	1.00	3.4
2:1 Alginate: Collagen 25°C	1.1E-02	2.9	0.8	1.00	3.5
2:1 Alginate: Collagen 35°C	4.2E-06	2.7	0.7	1.00	4.0
2:1 Alginate: Collagen 45°C	2.5E-14	1.8	0.8	1.00	2.8
1:1 Alginate: Collagen 25°C	6.4E+00	2.7	0.7	1.00	2.6
1:1 Alginate: Collagen 35°C	4.5E+00	3.2	0.6	1.00	2.1
1:1 Alginate: Collagen 45°C	5.5E+00	1.4	0.8	0.98	2.6

Citation: Zimmerling, A.; Zhou, Y.; Chen, X. Synthesis of Alginate/Collagen Bioink for Bioprinting Respiratory Tissue Models. *J. Funct. Biomater.* **2024**, *15*, 90. https://doi.org/10.3390/jfb15040090

Academic Editor: Dennis Douroumis

Received: 26 February 2024
Revised: 17 March 2024
Accepted: 29 March 2024
Published: 1 April 2024

Copyright: © 2024 by the authors. Licensee MDPI, Basel, Switzerland. This article is an open access article distributed under the terms and conditions of the Creative Commons Attribution (CC BY) license (https://creativecommons.org/licenses/by/4.0/).

Disclaimer/Publisher's Note: The statements, opinions and data contained in all publications are solely those of the individual author(s) and contributor(s) and not of MDPI and/or the editor(s). MDPI and/or the editor(s) disclaim responsibility for any injury to people or property resulting from any ideas, methods, instructions or products referred to in the content.
